# The Role of Perceived Organizational Support in Mental Health of Healthcare Workers During the COVID-19 Pandemic: A Cross-Sectional Study

**DOI:** 10.3389/fpsyt.2021.707293

**Published:** 2021-11-01

**Authors:** Andreas Chatzittofis, Anastasia Constantinidou, Artemios Artemiadis, Kyriaki Michailidou, Maria N. K. Karanikola

**Affiliations:** ^1^Medical School, University of Cyprus, Nicosia, Cyprus; ^2^Department of Clinical Sciences and Psychiatry, Umeå University, Umeå, Sweden; ^3^Biostatistics Unit, Cyprus School of Molecular Medicine, The Cyprus Institute of Neurology and Genetics, Nicosia, Cyprus; ^4^Department of Nursing, School of Health Sciences, Cyprus University of Technology, Limassol, Cyprus

**Keywords:** COVID-19, post-traumatic stress, depression, healthcare workers, organizational support

## Abstract

**Background:** Data support the link between the coronavirus disease 2019 (COVID-19) pandemic and mental distress in healthcare workers (HCWs). Although previous studies have documented the association between organizational policies and employees' psychological and mental status, there is still scant evidence regarding the effect of perceived organizational support (POS) on mental distress in HCWs during the pandemic.

**Aims:** The present study aimed to assess the association between POS and mental distress in HCWs during the COVID-19 pandemic. The role of POS in stress, depressive and trauma symptoms in HCWs was investigated.

**Methods:** This was an online cross-sectional study in 424 HCWs. Data were collected during the first wave of the pandemic, and included demographics, a 7-item questionnaire assessing POS, the “Patient Health Questionnaire” assessing depressive symptoms, the “Impact of Events Scale Revised,” measuring post-traumatic stress disorder (PTSD) symptoms and the “Perceived Stress Scale” assessing perceived stress.

**Results:** The mean POS score was 3.33 [standard deviation:1.85; range 0–7]. Younger (*p* < 0.001), less experienced (*p* < 0.001), female (*p* = 0.002), and non-physician HCWs (*p* = 0.031) were more likely to report lower self-perceived organizational support than older, male, more experienced physicians. Self-perceived organizational support was significantly and negatively associated with and self-assessed intensity of stress, depressive and traumatic symptoms, after adjusting for putative confounders (*p* < 0.001).

**Discussion:** Self-perceived organizational support was significantly associated with HCWs' self-assessed mental status during the pandemic. Organizational support and mental distress should be addressed simultaneously in HCWs during the COVID-19 pandemic to increase resilience among them.

## Introduction

Since the initial outbreak of the coronavirus disease (COVID-19) pandemic, reports have highlighted the impact of the pandemic on HCWs' mental and psychological health (1). Indeed, an increased frequency of psychiatric symptoms ranging between 11 and 75% has been reported in HCWs during previous relevant crises. A number of personal, work-related and organizational factors have been identified as risk factors for developing psychiatric symptoms in employees (2, 3).

Perceived organizational support (POS) refers to employees' perception regarding the extent to which their organization takes measures to protect their physical and psychological well-being (4). Additionally, POS has many implications as it is related to job satisfaction, organizational performance and absenteeism (5). However, there is limited pertinent research during health crises, such as the COVID-19 pandemic. Specifically, only a few studies have explored organizational factors in relation to mental health outcomes during the pandemic, including dimensions of organizational support to HCWs (6–9). These dimensions included education in self-protection, provision of protective equipment and psychological support and participation in decision making (6). A recent review pointed out the heterogeneity regarding the psychological and organizational measures used, as well as their cultural context; however, these findings support the association between organizational characteristics and mental health status of employees (10). Moreover, previous studies were conducted in specific cultural context, thus jeopardizing the generalization of their findings (11, 12). Overall, there are only a few empirical studies on the specific effects of POS on HCWs' mental health, and especially on different types of symptoms such as post-traumatic and depressive symptoms, or perceived stress.

Regarding COVID-19 context, recent studies have reported high levels of depressive and post-traumatic symptoms in up to 30% of HCWs (13). A challenging work environment, characterized by increased work demands and lack of organizational or colleague support may be linked to deterioration of mental and physical health in HCWs (6, 11, 12). Risk factors for developing these symptoms include personal history of mental disorders, longer work experience, older age, and adjustment difficulties (14). Most importantly, mental and psychological distress in HCWs has been associated with poor quality of care, less productivity and increased risk for errors (15). Thus, it becomes important for health organizations to identify the organizational needs of HCWs and to ascertain the impact of organizational aspects on their employees' mental health (16). So far, the majority of studies investigating risk factors related to adverse mental health outcomes in HCWs has mainly focused on personal factors such as occupation, sex, proximity of working with COVID-19 patients and history of mental health disorders, e.g., depression (13, 17).

Despite the unprecedented situations and needs created in healthcare systems by the pandemic, evidence on the interventional strategies for protecting HCW's mental health is still scarce (18). Healthcare systems are still in the process of understanding the problem, which forestalls the implementation of interventional policies (18).

The aim of this study was to shed more light on the relationship of POS with depressive and post-traumatic symptoms, and perceived stress in HCWs during the COVID-19.

## Materials and Methods

This was an online cross-sectional study. Data collection took place during the first wave of the COVID-19 pandemic (3–27 of May), just before the start of the gradual easing of restrictions following the first lockdown, in the Republic of Cyprus (RC). The questionnaire was disseminated through national professional associations (Medical, Physiotherapists) as well as through targeted social networks to nurses, occupational therapists, physicians and pharmacists. Informed consent for participation in the study was given through the web-based platform. The study protocol was approved by the National Bioethics Committee (number: 2020.01.89), and is described in more detail elsewhere (17).

The data collection tool included demographic, self-assessed psychological distress variables and a 7-item descriptive questionnaire on POS characteristics, developed by the authors according to literature (2) (Table 1). Each question was answered by No (0 point)/Yes (1). (Total scores of the perceived organizational support questionnaire range 0–7 points).

**Table 1:** Perceived organizational support (POS) questionnaire.

**Number**	**Items included in the questionnaire**
(1)	Do you think you are adequately prepared to provide care to patients with COVID-19?
(2)	Do you think you have all necessary means (Personal Protective Equipment) to protect yourself against COVID-19 at your workplace?
(3)	Do you think that all necessary protective measures against COVID-19 have been applied to protect you from transmitting COVID-19 to your family by your organization (e.g., rapid testing, isolation measures)?
(4)	Do you have access to psychological support services in case you need it?
(5)	Would you say that you adequately participate in decision-making regarding the pandemic at your workplace?
(6)	Do you have any kind of support regarding your personal needs? (e.g., balanced nutrition, hydration, rest, communication with your family)?
(7)	Do you think that you will get adequate support for yourself and family in case you have to be in quarantine due to COVID-19 infection?

The 9-item Patient Health Questionnaire (PHQ-9) was used for assessing depressive symptoms (Items are scored 0–3, scale score range 0–27, with higher scores corresponding to more severe symptoms of depression) (19).

The 22-item Impact of Events Scale Revised (IES-R) was used for assessing post-traumatic stress symptoms during the last 7 days (items are scored 0–4, scale score range 0–88, with higher scores corresponding to more severe symptoms of post-traumatic stress) (20).

The 10-item Perceived Stress Scale (PSS-10) was used for assessing self-perceived stress (items are scored 0–4, scale score range 0–40, with higher scores reflecting higher perceived stress levels) (21).

The online questionnaire did not allow for missing values, since giving an answer was obligatory to move to the next question and submit the questionnaire. As a result, missing data and possible bias was avoided. Additionally, aiming to minimize selection bias, the questionnaire was disseminated through national professional associations, ensuring access to all healthcare professionals in the RC.

Since this is a cross-sectional study, multivariate analysis was performed to address the main study aim and the sample size was a priori calculated accordingly by using G^*^Power software. A total of 416 individuals were needed, given a small to medium effect size of f2 = 0.06, an alpha error of 0.05, a power of 95% with about 10 predictors in the final model.

### Data Analysis

Descriptive statistics of demographic and clinical variables were reported as mean (M) and standard deviations (SD), or frequencies for continuous and categorical variables, respectively. The overall scores of the PHQ-9, IES-R, PSS-10 and POS scales were calculated as the sum of component items' scores. Correlations between the POS score and the total score of the PHQ-9, IES-R, and PSS-10 scales, respectively, were assessed using the Pearson's correlation coefficient (rho). We also performed multivariate linear regression analyses to test the significance of POS as predictor of IES-R, PHQ-9 and PSS-10 scores as dependent variables, after adjusting for putative confounders. Adjusting variables for the regression model were selected based on univariate analyses and only variables that were statistically significant were further included in the regression models. More specifically, multiple univariate analyses were performed between the different study variables and each mental health-related outcome. The putative confounders were then entered in multivariate models and checked for their multicollinearity. Variables showing increased multicollinearity were deleted from the final analysis. For brevity issues, this analysis is not presented, and the final confounders are only mentioned as footnotes in the results section. Statistical significance was set at *p* < 0.05, and all tests were 2-tailed. Data analyses was performed using SPSS-22 (Armonk, NY: IBM Corp).

## Results

### Participants' Demographics and Clinical Characteristics

A total of 424 HCWs with a mean of 13.1 years of work experience participated in the study. Two-hundred forty-eight (58.5%) were female and 176 (41.5%) were male, with a mean age of 38.8 years. One hundred seventy-eight (42%) were physicians, 57 (13.4%) had a positive history of depression or/and anxiety disorder. The demographics, occupational and clinical characteristics of participants are presented in Table 2.

**Table 2:** Participants' demographics, occupational variables, and clinical characteristics (*N* = 424).

**Numeric variable**	**Mean ± SD (range)**
Age (years)	38.8 ± 11.4 (21–76)
Work experience (years)	13.1 ± 10.2 (0–50)
**Categorical variable**	**n (%)**
Sex	
Males	176 (41.5)
Females	248 (58.5)
Occupation	
Physician	178 (42)
Nurse	103/ (24)
Other	143/ (34)
Work sector	
Inpatient public	193 (45.5)
Inpatient private	67 (15.8)
Outpatient private	142 (33.5)
Emergency	8 (1.9)
COVID-19 unit	14 (3.3)
COVID-19 care giving	
No	244 (57.5)
Yes	180 (42.5)
Married	
Yes	241 (56.8)
No	183 (43.2)
Parenthood	
Yes	239 (56.6)
No	183 (43.4)
Living alone	
Yes	79 (18.6)
No	345 (81.4)
Smoking	
Yes	124 (29.2)
No	300 (70.8)
Personal history of anxiety/depression	
Yes	57 (13.4)
No	367 (86.6)
Work status (currently working)	
Yes	394 (92.9)
No	30 (7.1)

### POS and Mental Distress of Participants

The mean score in the POS questionnaire was 3.33 [standard deviation (SD):1.85; range 0–7]; this was negatively correlated with IES-R, PSS-10 and PHQ-9 scores (*r* = −0.29; *r* = −0.289; *r* = −0.278, respectively, *p* < 0.001 for all correlations).

Males compared to females (3.7 ± 1.9 vs. 3.13.1 ± 1.8; *p* = 0.002) and physicians compared to nurses/other professionals (3.6 ± 1.7 vs. 3.2 ± 1.9; *p* = 0.031) reported higher POS score. Physicians compared to nurses (3.6 ± 1.7 vs. 2.8 ± 2.0; *p* = 0.001) reported, also, higher POS score. Participants who were directly involved in COVID-19 patient care reported lower POS score compared to those who were not (3.1 ± 1.8 vs. 3.5 ± 1.8; *p* = 0.011).

POS score was also positively correlated with age (*r* = 0.184; *p* < 0.001) and years of work experience (*r* = 0.160; *p* < 0.001).

### Regression Analyses Investigating the Association Between Organizational Support and Mental Distress

Linear regression analyses showed a significant negative relationship between perceived organizational support and self-assessed mental distress (i.e., symptoms of post-traumatic stress, depression, and stress), after adjusting for putative confounders (Table 3). In total, POS score explained 3.5–4.4% of the variability of mental distress in HCWs.

**Table 3:** Linear regression models on the role of perceived organizational support score (dependent variable) on IES, PSS, and PHQ scores, after adjusting for multiple confounders (*N* = 424).

**Regression model**	**B**	**SE**	**Total R^**2**^(%)**	**ΔR^**2**^(%)**	**Sig. (change)**	**Sig**.
IES-R^a^	−1.70	0.35	18.5	+4.4	<0.001^*^	<0.001^*^
PSS-10^b^	−0.75	0.17	24.6	+3.5	<0.001^*^	<0.001^*^
PHQ-9^c^	−0.56	0.12	16.2	+3.9	<0.001^*^	<0.001^*^

a *Adustment factors: Age, gender, Children, Occupation, COVID-19 caregiving, personal history of anxiety, or depression diagnosis*.

b *Adustment factors Age, gender, marital status, occupation, COVID-19 caregiving, personal history of anxiety, or depression diagnosis*.

c *Adustment factors Age, gender, marital status, occupation, COVID-19 caregiving, personal history of anxiety, or depression diagnosis*.

* *p < 0.05*.

## Discussion

In this study, we explored the association between POS and mental distress in HCWs during the first wave of the pandemic. Analyses showed that younger, less experienced, and non-physician females were more likely to report lower POS than older, more experienced, male physicians. Lower POS was also significantly associated with mental distress in terms of depressive and post-traumatic symptoms, and perceived stress, even after adjusting for multiple confounders. Although, the overall variance explained by the models, seems rather small, the finding that perceived organizational support, has an impact on mental health, is very important. Especially, as previous research on the influence of environmental factors on mental health outcomes show similar or lower effect sizes. This is due to the complex relationship between mental health and environmental factors, including many mediators and moderators such as genetics, epigenetics and other possible confounders.

Strengths of the present study include the timing of data collection, i.e., at the peak of the pandemic, the assessment of depressive, PTSD and stress symptoms simultaneously, and measurement of both individual and organizational risk factors. Moreover, the sampling method applied herein supports the internal validity of the present results. However, although the questionnaire was disseminated to all HCWs, possible selection bias cannot be excluded. Limitations include its cross-sectional design not allowing etiological inferences, the self-assessment of the HCWs' mental health, and possible cultural particularities; since this study was conducted in a specific geographic area (Cyprus), additional studies are needed to replicate these results. Moreover, additional confounders may have contributed to the mental distress reported herein which were not taken into consideration, such as fear of a COVID-19 infection, personality traits and medical history. Additionally, POS dimensions such as organizational policies and procedures, or workplace training and supervision, were not included in the present analyses. Finally, although the POS questionnaire was not validated prior to its use, it was based on a scoping review focused on HCWs' needs during the pandemic (6).

Nevertheless, the present study is among the few addressing organizational support in relation to mental distress in HCWs (7–9). A study in healthcare providers in Jordan revealed a link between burnout symptoms and inadequate personal protective equipment, limited access to COVID-19 testing and lack of measures to prevent transmission of COVID-19 to family members (9). Overall, POS provided by hospital managers has been highlighted as the strongest protective factor against depressive, post-traumatic and anxiety symptoms in frontline HCWs (8). The study by Zhang et al. (7), identified three organizational factors, namely “work support,” “personal support,” and “risk support,” which were negatively associated with anxiety, whereas “work support” and “personal support” predicted higher life satisfaction in HCWs. A study in Ethiopia reported that protection and support of the needs of HCWs is a crucial factor toward their engagement to work, and provision of optimal care to patients (22).

Importantly, in a recent review female nurses working in intensive care units and emergency departments reported the highest levels of burnout, anxiety and depressive symptoms during the pandemic compared to other healthcare professionals (1, 23). Work environment, communication, and support by supervisors were identified as important risk factors regarding mental health symptoms.

Regarding work-related PTSD symptoms, the present results are in line with the literature reporting that organizational factors such as the heavy workload, young age, female gender, lack of training and support are important predictors of PTSD symptoms in HCWs during the pandemic (14, 24). Moreover, it has been proposed that hospital managers should anticipate the impact of the pandemic on the mental health of vulnerable HCWs, i.e., females and frontline workers, by implementing educational sessions on coping with stressful events and developing resilience (14, 25). These evidences altogether outline the importance of data on POS regarding the development of supportive measures toward mental health of HCWs.

The importance of organizational support on HCWs' wellbeing has been clearly evident during the pandemic; this crisis brought to the spotlight fundamental differences between HCWs and administrators in prioritizing measures (26). Recently, a package of recommendations on how to build resilience in HCWs, prior and during an epidemic outbreak, was published suggesting interventions at both individual and organizational level (27). However, only a few hospitals around the world have developed protocols to support mental health in HCWs during the pandemic; most of relevant interventions have been implemented at individual level, e.g., group or personal supportive sessions, instead of addressing organizational empowerment (28, 29). Additionally, the majority of healthcare organizations provide organizational support to nurses by covering basic needs, such as food, childcare, mental health support and COVID-19 testing, rather than addressing other types of support, such as participation in decision-making (30). Shah et al. (31), suggested that cultivation of a transparent, open-ended mode of communication, especially in leadership, and a supportive work environment is expected to increase resilience in HCWs during and after the pandemic (31). Healthcare administrators collaboratively with HCWs are expected to develop and implement supportive programs toward employees during crisis events, including the COVID-19 pandemic.

Social support by colleagues and managers and a positive workplace climate has positive effects on perceived psychological distress. Therefore, targeted interventions can counteract the effects of work-related stress (32). It has been shown that ethical leadership can alleviate the perception of work-related stress and improve the quality of the relationship between supervisors and subordinates (33). In addition, even the supervisors' POS is very important, as it affects their relationships with the subordinates, their job satisfaction and their job performance (34).

In conclusion, these findings highlight the importance of POS on the mental health of HCWs during periods of crisis such as the COVID-19 pandemic; direct implications to policy makers and administrators to increase resilience in HCWs through the development of comprehensive supportive strategies are supported by the present findings. As mental distress including depression and PTSD are risk factors for psychological impairment and suicidal behavior, specific and urgent preventive measures should be implemented (24), along with regular screening of psychiatric symptoms in HCWs (24). Moreover, since PTSD symptoms may develop later in the course of the pandemic, longitudinal studies are proposed.

## Data Availability Statement

The datasets presented in this article are not readily available due to restrictions from the Cyprus National Bioethics Committee. Requests to access the datasets should be directed to chatzittofis.andreas@ucy.ac.cy.

## Ethics Statement

The participants provided their online informed consent to participate in this study.

## Author Contributions

ACh, ACo, and MK conceptualized the study. ACh was responsible for the data collection, coordination and supervision of the data collection. AA, KM, and ACh carried out the statistical analyses. ACh and AA wrote the first draft of the manuscript. MK also participated in writing the final manuscript. All authors were responsible for the design of the study, responsible for the interpretation of the data, and revised the paper critically or commented on important issues and gave final approval of the version to be published.

## Conflict of Interest

The authors declare that the research was conducted in the absence of any commercial or financial relationships that could be construed as a potential conflict of interest.

## Publisher's Note

All claims expressed in this article are solely those of the authors and do not necessarily represent those of their affiliated organizations, or those of the publisher, the editors and the reviewers. Any product that may be evaluated in this article, or claim that may be made by its manufacturer, is not guaranteed or endorsed by the publisher.

## References

[1] Gualano MR Sinigaglia T Lo Moro G Rousset S Cremona A Bert F. The burden of burnout among healthcare professionals of intensive care units and emergency departments during the COVID-19 pandemic: a systematic review. Int J Environ Res Public Health. (2021) 18:8172. 10.3390/ijerph18158172 34360465 PMC8346023

[2] Preti E Di Mattei V Perego G Ferrari F Mazzetti M Taranto P. The psychological impact of epidemic and pandemic outbreaks on healthcare workers: rapid review of the evidence. Curr Psychiatry Rep. (2020) 22:43. 10.1007/s11920-020-01166-z 32651717 PMC7350408

[3] Carmassi C Foghi C Dell'Oste V Cordone A Bertelloni CA Bui E. PTSD symptoms in healthcare workers facing the three coronavirus outbreaks: what can we expect after the COVID-19 pandemic. Psychiatry Res. (2020) 292:113312. 10.1016/j.psychres.2020.113312 32717711 PMC7370915

[4] Sun L. Perceived organizational support: a literature review. (2019). Int J Hum Resour Stud 9:21. 10.5296/ijhrs.v9i3.1510212184574

[5] Rhoades L Eisenberger R. Perceived organizational support: a review of the literature. J Appl Psychol. (2002) 87:698–714. 10.1037/0021-9010.87.4.698 12184574

[6] Shanafelt T Ripp J Trockel M. Understanding and addressing sources of anxiety among health care professionals during the COVID-19 pandemic. JAMA. (2020) 323:2133–4. 10.1001/jama.2020.5893 32259193

[7] Zhang SX Sun S Afshar Jahanshahi A Alvarez-Risco A Ibarra VG Li J. Developing and testing a measure of COVID-19 organizational support of healthcare workers - results from Peru, Ecuador, and Bolivia. Psychiatry Res. (2020) 291:113174. 10.1016/j.psychres.2020.113174 32585436 PMC7284268

[8] Feingold JH Peccoralo L Chan CC Kaplan CA Kaye-Kauderer H Charney D. Psychological impact of the COVID-19 pandemic on frontline health care workers during the pandemic surge in New York City. Chronic Stress. (2021) 5:2470547020977891. 10.1177/2470547020977891 33598592 PMC7863176

[9] Algunmeeyn A El-Dahiyat F Altakhineh MM Azab M Babar ZUD. Understanding the factors influencing healthcare providers' burnout during the outbreak of COVID-19 in Jordanian hospitals. J Pharm Policy Pract. (2020) 13:53. 10.1186/s40545-020-00262-y 32974035 PMC7505678

[10] Giorgi G Lecca LI Alessio F Finstad GL Bondanini G Lulli LG. COVID-19-related mental health effects in the workplace: a narrative review. Int J Environ Res Public Health. (2020) 17:7857. 10.3390/ijerph17217857 33120930 PMC7663773

[11] Liu D Baumeister RF Veilleux JC Chen C Liu W Yue Y. Risk factors associated with mental illness in hospital discharged patients infected with COVID-19 in Wuhan, China. Psychiatry Res. (2020) 292:113297. 10.1016/j.psychres.2020.113297 32707218 PMC7355324

[12] Martin-Delgado J Viteri E Mula A Serpa P Pacheco G Prada D. Availability of personal protective equipment and diagnostic and treatment facilities for healthcare workers involved in COVID-19 care: A cross-sectional study in Brazil, Colombia, and Ecuador. PLoS ONE. (2020) 15:e0242185. 10.1371/journal.pone.0242185 33175877 PMC7657544

[13] Serrano-Ripoll MJ Meneses-Echavez JF Ricci-Cabello I Fraile-Navarro D Fiol-deRoque MA Pastor-Moreno G. Impact of viral epidemic outbreaks on mental health of healthcare workers: a rapid systematic review and meta-analysis. J Affect Disord. (2020) 277:347–57. 10.1016/j.jad.2020.08.034 32861835 PMC7443314

[14] D'Ettorre G Pellicani V Ceccarelli G. Post-traumatic stress disorder symptoms in healthcare workers: a ten-year systematic review. Acta Biomed. (2020) 91:e2020009. 10.23750/abm.v91i12-S.9459 33263341 PMC8023102

[15] Karanikola MNK Zartaloudi A Nystazaki M Zavrou R Papathanassoglou EDE. Is there any association among depressive symptoms, job satisfaction and self-assessed empathy? A correlational study in Greek Psychiatric/Mental Health Nurses. Arch Psychiatr Nurs. (2020) 34:230–6. 10.1016/j.apnu.2020.04.006 32828354

[16] Nagesh S Chakraborty S. Saving the frontline health workforce amidst the COVID-19 crisis: challenges and recommendations. J Glob Health. (2020) 10:010345. 10.7189/jogh.10.010345 32373323 PMC7183244

[17] Chatzittofis A Karanikola M Michailidou K Constantinidou A. Impact of the COVID-19 pandemic on the mental health of healthcare workers. Int J Environ Res Public Health. (2021) 18:1435. 10.3390/ijerph18041435 33546513 PMC7913751

[18] Pollock A Campbell P Cheyne J Cowie J Davis B McCallum J. Interventions to support the resilience and mental health of frontline health and social care professionals during and after a disease outbreak, epidemic or pandemic: a mixed methods systematic review. Cochrane Database Syst Rev. (2020) 11:CD013779. 10.1002/14651858.CD013779 33150970 PMC8226433

[19] Hyphantis T Kotsis K Voulgari PV Tsifetaki N Creed F Drosos AA. Diagnostic accuracy, internal consistency, and convergent validity of the Greek version of the patient health questionnaire 9 in diagnosing depression in rheumatologic disorders. Arthritis Care Res (Hoboken). (2011) 63:1313–21. 10.1002/acr.20505 21618450

[20] Mystakidou K Tsilika E Parpa E Galanos A Vlahos L. Psychometric properties of the impact of event scale in Greek cancer patients. J Pain Symptom Manage. (2007) 33:454–61. 10.1016/j.jpainsymman.2006.09.023 17397706

[21] Andreou E Alexopoulos EC Lionis C Varvogli L Gnardellis C Chrousos GP. Perceived stress scale: reliability and validity study in Greece. Int J Environ Res Public Health. (2011) 8:3287–98. 10.3390/ijerph8083287 21909307 PMC3166743

[22] Zewudie A Regasa T Kebede O Abebe L Feyissa D Ejata F. Healthcare professionals' willingness and preparedness to work during COVID-19 in selected hospitals of southwest Ethiopia. Risk Manag Healthc Policy. (2021) 14:391–404. 10.2147/RMHP.S289343 33568957 PMC7868776

[23] Sun P Wang M Song T Wu Y Luo J Chen L. The psychological impact of COVID-19 pandemic on health care workers: a systematic review and meta-analysis. Front Psychol. (2021) 12:626547. 10.3389/fpsyg.2021.626547 34305703 PMC8297953

[24] d'Ettorre G Ceccarelli G Santinelli L Vassalini P Innocenti GP Alessandri F. Post-traumatic stress symptoms in healthcare workers dealing with the COVID-19 pandemic: a systematic review. Int J Environ Res Public Health. (2021) 18:601. 10.3390/ijerph18020601 33445712 PMC7828167

[25] Zhou JY Norman AW Chen DL Sun GW Uskokovic M Koeffler HP. 1,25-Dihydroxy-16-ene-23-yne-vitamin D3 prolongs survival time of leukemic mice. Proc Natl Acad Sci USA. (1990) 87:3929–32. 10.1073/pnas.87.10.3929 2339131 PMC54017

[26] Mullangi S Diamond R Patel KK. Bridging the divide between physicians and administrators during COVID-19. Am J Manag Care. (2020) 26:499–500. 10.37765/ajmc.2020.88497 33315323

[27] Rieckert A Schuit E Bleijenberg N Ten Cate D De Lange W De Man-Van Ginkel JM. How can we build and maintain the resilience of our health care professionals during COVID-19? Recommendations based on a scoping review. BMJ Open. (2021) 11:e043718. 10.1136/bmjopen-2020-043718 33408212 PMC7789206

[28] Buselli R Corsi M Veltri A Baldanzi S Chiumiento M Lupo ED. Mental health of Health Care Workers (HCWs): a review of organizational interventions put in place by local institutions to cope with new psychosocial challenges resulting from COVID-19. Psychiatry Res. (2021) 299:113847. 10.1016/j.psychres.2021.113847 33721785 PMC7920813

[29] Morrow E Call M Ransco M Hofmann KM Locke A. Sustaining workforce well-being: a model for supporting system resilience during the COVID-19 pandemic. Glob Adv Health Med. (2021) 10:2164956121991816. 10.1177/2164956121991816 33708458 PMC7907930

[30] Cho H Sagherian K Steege LM. Hospital nursing staff perceptions of resources provided by their organizations during the COVID-19 pandemic. Workplace Health Saf. (2021) 69:174–81. 10.1177/2165079920987543 33514301 PMC7862911

[31] Shah M Roggenkamp M Ferrer L Burger V Brassil KJ. Mental health and covid-19 the psychological implications of a pandemic for nurses. Clin J Oncol Nurs. (2021) 25:69–75. 10.1188/21.CJON.69-75 33480882

[32] Lecca LI Finstad GL Traversini V Lulli LG Gualco B Taddei G. The role of job support as a target for the management of work-related stress: the state of art. Qual Access Success. (2020) 21:152–8. Available online at: https://www.proquest.com/docview/2350963867?pq-origsite=gscholar&fromopenview=true

[33] Zhou H Jin M Ma Q. Remedy for work stress: the impact and mechanism of ethical leadership. Cent Eur J Public Health. (2015) 23:176–80. 10.21101/cejph.a4246 26851431

[34] Erdogan B Enders J. Support from the top: supervisors' perceived organizational support as a moderator of leader-member exchange to satisfaction and performance relationships. J Appl Psychol. (2007) 92:321–30. 10.1037/0021-9010.92.2.321 17371081

